# The Academic Hunger Gap: From Plates to Well‐Being—How Food Insecurity Undermines Quality of Life in University Students

**DOI:** 10.1002/fsn3.71663

**Published:** 2026-03-16

**Authors:** Ümmühan İnci Kandemir, Süleyman Utku Uzun

**Affiliations:** ^1^ Department of Public Health, Medical Faculty Pamukkale University Denizli Turkey; ^2^ Epidemiology Division, Department of Public Health, Medical Faculty Pamukkale University Denizli Turkey

**Keywords:** food insecurity, household food insecurity access scale (HFIAS), quality of life, short form‐12 (SF‐12), university students

## Abstract

Food insecurity, a significant global challenge, is increasingly recognized among university students, potentially impacting their quality of life (QoL). This study aimed to assess the prevalence of food insecurity and its association with QoL among university students in Denizli, Türkiye. A cross‐sectional study was conducted among 1495 university students. Food insecurity was assessed using the Household Food Insecurity Access Scale (HFIAS), categorized as food secure, mild, moderate, or severe insecurity. Quality of life was measured with the SF‐12, yielding Physical Component Summary (PCS) and Mental Component Summary (MCS) scores. Multiple linear regression analyses were performed to identify predictors of physical (PCS) and mental (MCS) components of quality of life. The prevalence of food insecurity was 44.9%, with 20.9% experiencing mild, 16.0% moderate, and 8.0% severe food insecurity. Food‐insecure students had significantly lower PCS (50.16 ± 8.05 vs. 52.50 ± 6.89, *p* < 0.001) and MCS scores (38.24 ± 10.30 vs. 41.58 ± 10.28, *p* < 0.001) compared to food‐secure students. A dose–response was observed; increasing food insecurity severity correlated with decreasing PCS and MCS scores (*p* < 0.001 for both). In multivariable models, food insecurity remained an independent negative predictor of PCS and MCS, alongside other factors such as income, chronic disease, smoking, and gender. Food insecurity affects nearly half of university students and is significantly associated with poorer physical and mental quality of life, even after controlling for various confounding factors. These findings highlight the urgent need for targeted university interventions including campus food assistance programs, enhanced financial aid policies to address food insecurity and improve student well‐being.

## Graphical abstract

This cross‐sectional study of 1495 university students at Pamukkale University in Türkiye reveals that 44.9% of the study population experiences food insecurity. A clear dose–response relationship was observed: as the severity of food insecurity increases (mild, moderate, to severe), both physical and mental quality of life significantly decline. Food insecurity serves as an independent predictor of lower well‐being, highlighting an urgent call to action for campus food support, routine screening, and financial aid.

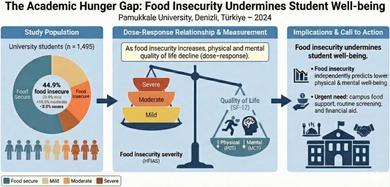

## 1 Introduction

The concept of “quality of life” (QoL) is a broad and multidimensional construct that has been defined in various ways across academic disciplines. A widely accepted definition comes from the World Health Organization (WHO), which describes QoL as “an individual's perception of their position in life in the context of the culture and value systems in which they live and in relation to their goals, expectations, standards and concerns” (WHO 1998). Nutritional adequacy—a cornerstone of food security—plays a critical yet understudied role in shaping QoL.

Food insecurity—defined as limited or uncertain access to nutritionally adequate and socially acceptable food—is a major social determinant of health and is increasingly recognized as relevant to quality of life outcomes (United States Department of Agriculture Economic Research Service 2014). Global food insecurity remains a critical challenge, with approximately 713 to 757 million people facing hunger in 2023 (Food and Agriculture Organization 2024). In Türkiye, this issue is also prevalent; the 2019 Turkey Nutrition and Health Survey reported that nearly a quarter of the population experiences anxiety about food access or lacks dietary diversity (Republic of Turkey Ministry of Health, General Directorate of Public Health 2019).

Food insecurity's ramifications extend beyond caloric deprivation, permeating physical and mental health domains. Nutritionally inadequate diets weaken immune responses, elevate risks of non‐communicable diseases (e.g., diabetes, hypertension), and correlate with micronutrient deficiencies (World Health Organization 2003). A broader systematic review and meta‐analysis investigating the link between food insecurity and psychological distress found a strong positive association, indicating that individuals experiencing food insecurity are more likely to suffer from depression, anxiety, and sleep problems, and report lower life satisfaction (Jandaghian‐Bidgoli et al. 2024).

According to the 2023 Global Hunger Index Report, young people are an important but often overlooked group disproportionately affected by crises and food insecurity (von Grebmer et al. 2023). University students—a transitional population navigating financial independence and academic pressures—are a uniquely vulnerable group with respect to food insecurity. The transition to higher education is frequently accompanied by financial constraints due to elevated educational costs, limited income sources, and the burdens of managing a rigorous academic schedule (El Zein et al. 2019). Factors such as high tuition fees, the costs of textbooks and accommodation, and the scarcity of time to prepare healthy meals can compel students to opt for cheaper, yet nutritionally inadequate, food options (Dharmayani et al. 2024; Lemp et al. 2023; Shi et al. 2021). Consequently, these challenges not only impair students' nutritional intake but also exacerbate stress and adversely affect their academic performance and overall quality of life (Betancourt‐Núñez et al. 2024; Brownfield et al. 2023; Esin and Ayyıldız 2024).

Research on the specific relationship between food insecurity and quality of life among university students in Türkiye is relatively limited in scope and geographical coverage, suggesting a need for further investigation at both national and regional levels. This study aims to bridge this gap by evaluating the impact of food insecurity on the quality of life among students at Pamukkale University in Denizli.

## 2 Methods

### 2.1 Study Design and Setting

This cross‐sectional study was conducted at Pamukkale University's central campus between 1 November and 31 December 2024. Data were collected from students enrolled in sixteen faculties: Dentistry, Education, Science, Physiotherapy & Rehabilitation, Law, Economics & Administrative Sciences, Theology, Communication, Humanities & Social Sciences, Architecture & Design, Engineering, Health Sciences, Sport Sciences, Technology, Medicine and Tourism.

### 2.2 Participants and Sampling

The sampling frame comprised all undergraduates registered in the 2024–2025 academic year (*N* = 45,698), of whom 29,282 attended the sixteen central‐campus faculties. Enrollment figures were obtained from the university's information system.

*Sample Size*: Using G*Power v3.1.9.7, we based our calculation on detected differences in SF‐12 quality‐of‐life scores between food‐secure and food‐insecure groups (Cohen's *d* = 0.20) (Cedillo et al. 2023; Hagedorn et al. 2021; Slotnick et al. 2024). Assuming *α* = 0.05 and power = 0.95, the minimum sample was 1302. Allowing for 15% nonresponse, the target sample was set at 1495.

*Sampling Method*: We employed convenience sampling with proportional allocation by faculty. Quotas were predetermined for each faculty based on their proportional share of the total central‐campus enrollment (proportional allocation). The target sample size (*n* = 1495) was set a priori based on the G*Power calculation and quotas were filled by approaching volunteering students until each faculty quota was met. At the end of data collection, we obtained 1495 fully completed questionnaires (the prespecified target). The number of students approached and the number of explicit refusals were not recorded during fieldwork; this is acknowledged as a study limitation.

### 2.3 Data Collection

Trained researchers administered paper surveys in classrooms and social areas (e.g., cafeterias, libraries…etc.). Before participation, students received information on study objectives, voluntariness, and confidentiality, and provided written informed consent. Each survey required approximately 10 min to complete; responses were anonymous.

### 2.4 Inclusion and Exclusion Criteria

Participants included students enrolled in central campus faculties during the study period and those providing voluntary consent. Exclusion criteria were refusal to participate or inability to complete the questionnaire due to language barriers (as the data collection form was in Turkish and administered under observation).

### 2.5 Instruments and Measures

#### 2.5.1 Questionnaire

A comprehensive questionnaire was developed after reviewing relevant literature. The questionnaire was self‐administered under observation. The questionnaire consisted of 45 items: 10 questions on sociodemographic characteristics, 1 question on smoking status, 1 question on chronic disease status, 2 questions on height and weight, 3 questions on source of financial support and academic performance, 9 items from the Household Food Insecurity Access Scale, 12 items from the SF‐12 Quality of Life Scale, 7 items from the International Physical Activity Questionnaire (short form).

*Demographics and Health Behaviors*: Age, gender, faculty, academic year, marital status, family structure, longest residence, parental education, family income, current residence, smoking status, chronic disease history, and self‐reported height/weight (for BMI calculation). Income was categorized into four levels based on self‐reported income status: (1) Insufficient income and in debt; (2) Insufficient income but no debt; (3) Sufficient income but no savings; (4) Sufficient income and able to save.

*Body Mass Index (BMI)*: Calculated as weight (kg)/[height (m)]^2^ and classified per WHO categories: underweight (< 18.5), normal (18.5–24.9), overweight (25.0–29.9), obese (30.0–39.9), and morbidly obese (≥ 40.0).

*Academic Achievement*: Self‐rated on a five‐point Likert scale (very good to very poor) and cumulative GPA.

### 2.6 Household Food Insecurity Access Scale (HFIAS)

The HFIAS was originally developed and validated to assess household food‐access insecurity (Coates et al. 2007). For this study, we used it to assess individual student‐level food insecurity experiences, as students represent individual economic units independent of their household situations. For this campus‐based study we adapted the occurrence questions to reflect student experiences within the university context and specified the recall period as during university life. The Turkish version of the “Household Food Insecurity Access Scale” (HFIAS) was adapted by Hakan Bor in 2018 (Bor 2018). The internal consistency of the HFIAS in our study was calculated with Cronbach's alpha value of 0.92, demonstrating good reliability.

The HFIAS consists of two types of complementary questions:

*Occurrence Questions*: Nine questions assessing whether specific food insecurity situations occurred during a specified period (modified to “during your university life” for this study similar to “since being in college” used by Hagedorn et al. 2021) to capture the cumulative or chronic dimensions of food insecurity experienced within the university context. Data were collected between 1 November and 31 December 2024; the sample therefore included first‐year students who had been enrolled for approximately 2–3 months as well as final‐year students. Because students' food access commonly varies between term‐time and vacation periods (e.g., students returning home during semester breaks, recent receipt of scholarships/loans, or parental support at term start), a short recall window (e.g., the last 30 days) could under‐ or over‐estimate experiences that are characteristic of the student's university life. We therefore used the longer “university life period” recall to focus responses on experiences attributable to university life rather than transient home circumstances. Response options were binary (0 = No, 1 = Yes). If a respondent answered “No”, they skipped the corresponding frequency question.

*Frequency Questions*: If an occurrence question was answered “Yes”, the respondent indicated how often the situation occurred, with three response options: 1 = Rarely, 2 = Sometimes, 3 = Often.

#### 2.6.1 HFIAS Scoring

Each frequency question was scored from 0 to 3, with occurrence questions answered “No” coded as 0. The total HFIAS score was calculated by summing all frequency question scores, ranging from 0 to 27. Higher scores indicated more severe food insecurity.

#### 2.6.2 Food Insecurity Categories

Total HFIAS scores were classified into four categories: 0–1: Food secure, 2–7: Mild food insecurity, 8–14: Moderate food insecurity, 15–27: Severe food insecurity.

### 2.7 Short Form‐12 (SF‐12) Quality of Life Scale

The SF‐12 is a self‐reported instrument evaluating the impact of health status on daily life (Ware et al. 1996). It is an abbreviated version of the SF‐36, originally developed in 1996. The scale assesses health status over the standard recall period of the past 4 weeks. The Turkish version's validity and reliability were established by Soylu and Kütük in 2022, with internal consistency coefficients of 0.73 and 0.72 for physical and mental components, respectively (Soylu and Kütük 2022).

The SF‐12 comprises 12 items across eight domains, grouped into two main components: Physical Health Component Summary (PCS) and Mental Health Component Summary (MCS). PCS includes the following domains: Physical Functioning (2 items), Physical Role Limitations (2 items), Bodily Pain (1 item), General Health (1 item). MCS includes the following domains: Vitality (Energy) (1 item), Social Functioning (1 item), Emotional Role Limitations (2 items), and Mental Health (2 items).

Items related to physical and emotional roles are answered as yes/no, while other items use Likert‐type responses ranging from 3 to 6 options. Scores are calculated for each subdomain using specific formulas and weightings, with no overall total score. Subscale scores range from 0 to 100, with higher scores indicating better health status and quality of life. There is no standard cut‐off point for these scores.

### 2.8 International Physical Activity Questionnaire (Short Form) (IPAQ)

The IPAQ Short Form consists of 7 questions assessing walking, moderate physical activities, and vigorous physical activities performed during the previous week. The calculation of total scores includes the duration (minutes) and frequency (days) of these activities, expressed as MET‐minutes. MET‐minutes: Physical activity was expressed as metabolic equivalent task minutes per week (MET‐minutes/week) representing the energy cost of physical activities. (MET value of activity × duration in minutes × days per week.) One MET‐minute is calculated by multiplying the activity minutes by the MET score (the baseline MET value for that specific activity). MET‐minute scores are based on kilocalorie values for a 60‐kg individual. Kilocalories are calculated using the equation: MET‐min × (individual's body weight in kg/60 kg). The following values are used for IPAQ data analysis: Walking = 3.3 METs, Moderate physical activity = 4.0 METs, Vigorous physical activity = 8.0 METs.

#### 2.8.1 IPAQ Categorical Classification

Physical activity levels are categorized into three levels as inactive, minimally active, and highly active per standard criteria.

The Turkish version of the IPAQ was adapted by Melda Öztürk in 2005 (Öztürk 2005).

### 2.9 Statistical Analysis

Analyses were performed with SPSS v29.0. Categorical variables are presented as frequencies and percentages; continuous variables as mean ± SD, median (IQR), and range. Normality was assessed via Kolmogorov–Smirnov tests, skewness/kurtosis (±1.96), coefficient of variation, and histograms. Surveys were administered under direct observation, ensuring complete responses; thus, there were no missing data. Independent samples *t*‐tests and one‐way ANOVA were used to compare mean PCS and MCS scores across food insecurity categories and other independent variables. Variables with *p* < 0.05 in univariate analyses were entered into a backward stepwise multiple linear regression model to identify independent predictors of SF‐12 scores. Significance was set at *p* < 0.05. Multicollinearity was checked using Variance Inflation Factors (VIF). All variables in the final models demonstrated VIF values < 3, indicating no substantial multicollinearity. Multiple linear regression (backward method) models were constructed to evaluate factors associated with quality of life. Variables with *p* < 0.05 in univariate analyses were included in the regression model.

### 2.10 Ethical Considerations

The protocol was approved by the Pamukkale University Non‐Interventional Clinical Research Ethics Committee (Approval No. E‐579242; Approval Date: 11 September 2024). All participants provided written informed consent, with assurances of anonymity. Permissions were obtained for all adapted scales.

## 3 Results

The study included 1495 university students with a mean age of 20.71 ± 3.40 years. A majority of participants were female (64.20%, *n* = 960). Parental education levels revealed that 32.60% (*n* = 487) of mothers had primary school education, while fathers generally had higher education levels, with 27.90% (*n* = 417) completing high school. In terms of perceived financial status, 43.10% (*n* = 644) reported having sufficient income but were unable to save. At the time of the survey, 42.20% (*n* = 631) lived in a state dormitory. Financial support for education primarily came from family (81.50%, *n* = 1218). In terms of health behaviors, 21.50% (*n* = 322) of the students reported currently smoking. Based on the Body Mass Index (BMI), 64.3% of the students were in the normal weight category, and 24.8% were above normal weight (20.1% overweight, 4.4% obese, 0.3% morbidly obese), with a mean BMI of 22.79 ± 3.99. A majority (52.4%, *n* = 783) were classified as highly active. The self‐reported academic achievement level was predominantly “moderate” (46.8%, *n* = 699). Sociodemographic and academic characteristics of students are presented in Table 1.

**TABLE 1:** Sociodemographic characteristics of participants.

Variables	Values	*n*	%
Age (mean ± SD)	20.71 ± 3.40		
Gender	Male	535	35.80
	Female	960	64.20
Faculty	Faculty of Economics and Administrative Sciences	307	20.50
	Faculty of Humanities and Social Sciences	271	18.10
	Faculty of Engineering	217	14.50
	Faculty of Education	141	9.40
	Faculty of Technology	90	6.00
	Faculty of Medicine	83	5.60
	Faculty of Architecture and Design	62	4.10
	Faculty of Tourism	57	3.80
	Faculty of Theology	49	3.30
	Faculty of Health Sciences	40	2.70
	Faculty of Science	39	2.60
	Faculty of Law	34	2.30
	Faculty of Sports Sciences	33	2.20
	Faculty of Dentistry	31	2.10
	Faculty of Communication	22	1.50
	Faculty of Physiotherapy and Rehabilitation	19	1.30
Academic year	1st year	438	29.30
	2nd year	603	40.30
	3rd year	269	18.00
	4th year	158	10.60
	6th year	27	1.80
Marital status	Married	24	1.60
	Single	1467	98.10
	Divorced/Widowed/Separated	4	0.30
Family type	Nuclear family	1237	82.70
	Extended family	157	10.50
	Separated parents	95	6.40
	Other	6	0.40
Longest place lived	City center	900	60.20
	District center	443	29.60
	Town/Village	152	10.20
Perceived income status	Income insufficient with debt	171	11.40
	Income insufficient without debt	168	11.20
	Income sufficient but no savings	644	43.10
	Income sufficient with savings	512	34.20
Current residence	State dormitory	631	42.20
	With family	444	29.70
	Student apartment/house (alone)	258	17.30
	Student apartment/house (with friends)	97	6.50
	Private dormitory	51	3.40
	With relatives/acquaintances	8	0.50
	Other	6	0.40
Mother's education level	Illiterate	42	2.80
	Literate	56	3.70
	Primary school graduate	487	32.60
	Middle school graduate	266	17.80
	High school graduate	371	24.80
	University graduate or higher	273	18.30
Father's education level	Illiterate	14	0.90
	Literate	22	1.50
	Primary school graduate	339	22.70
	Middle school graduate	290	19.40
	High school graduate	417	27.90
	University graduate or higher	413	27.60
Smoking status	Yes	322	21.50
	No	1121	75.00
	Quit	52	3.50
Chronic disease status	Yes	130	8.70
	No	1365	91.30
Financial support sources	Family	1218	81.50
	Government scholarship	267	17.90
	Educational/contribution loan	197	13.20
	Employment	188	12.60
	Private scholarship	59	3.90
	Other	27	1.80
Academic performance	Excellent	87	5.80
	Good	599	40.10
	Moderate	699	46.80
	Low	90	6.00
	Very low	20	1.30
GPA (mean ± SD)	70.55 ± 11.40		
Body Mass Index (BMI)	Underweight	160	10.80
	Normal weight	951	64.30
	Overweight	298	20.10
	Obese	65	4.40
	Morbidly obese	5	0.30
BMI (mean ± SD)	22.79 ± 3.99		
Physical activity level	Inactive	676	45.2
	Minimally active	36	2.4
	Highly active	783	52.4

*Note*: *N* = 1495 for all variables. GPA is reported on a 0–100 scale.

The median Household Food Insecurity Access Scale (HFIAS) score among participants was 1.00 (IQR 7.00) and scores ranging from 0 to 27. Overall, 44.90% (95% CI: 42.38–47.41) (*n* = 671) of students were classified as food insecure. Among those experiencing food insecurity, 20.9% (95% CI: 18.95–23.07) (*n* = 313) reported mild, 16.0% (95% CI: 14.22–17.93) (*n* = 239) moderate, and 8.0% (95% CI: 6.69–9.44) (*n* = 119) severe food insecurity.

Item‐level descriptive statistics for each SF‐12 question are provided in Table S1. Primary inferential analyses were conducted using the validated SF‐12 summary measures (PCS and MCS) calculated according to the standard scoring algorithm.

The SF‐12 Quality of Life scores demonstrated significant differences between food‐secure and food‐insecure students. Students experiencing food insecurity (50.16 ± 8.05) had significantly lower PCS scores compared to food‐secure students (52.50 ± 6.89) (*p* < 0.001). Similarly, students with food insecurity (38.24 ± 10.30) exhibited significantly lower MCS scores compared to their food‐secure counterparts (41.58 ± 10.28) (*p* < 0.001).

Further analysis based on the severity of food insecurity indicated a clear downward trend in both physical and mental scores as food insecurity worsened (*p* < 0.001 for both). Table 2 summarizes the distribution of food‐insecurity levels alongside SF‐12 physical and mental quality‐of‐life scores, stratified by both food‐insecurity status (secure vs. insecure) and severity (mild, moderate, severe) among students.

**TABLE 2:** Food insecurity levels, SF‐12 quality‐of‐life scores by food‐insecurity status and severity.

Quality of life	Food insecurity	*n* (%)	95% CI for %	Median (IQR)	Min–max	*p* ^a^
SF‐12, physical component score (PCS)	Overall	1495 (100%)	—	53.18 (10.03)	22.67–65.94	—
	*Food insecurity status*					
	Food secure	824 (55.10%)	52.59%–57.62%	54.20 (7.75)	24.77–65.94	< 0.001
	Food insecure	671 (44.90%)	42.38%–47.41%	51.59 (11.37)	22.67–65.78	
	*Severity of food insecurity*					
	Food secure	824 (55.10%)	52.59%–57.62%	54.20 (7.75)	24.77–65.94	< 0.001
	Mild food insecurity	313 (20.90%)	18.95%–23.07%	53.05 (9.64)	30.82–65.78	
	Moderate food insecurity	239 (16.00%)	14.22%–17.93%	51.02 (11.59)	22.67–65.58	
	Severe food insecurity	119 (8.00%)	6.69%–9.44%	47.68 (12.78)	22.69–64.70	
SF‐12, mental component score (MCS)	Overall	1495 (100%)	—	40.85 (15.54)	10.50–63.03	—
	*Food insecurity status*					
	Food secure	824 (55.10%)	52.59%–57.62%	42.49 (15.71)	11.40–63.03	< 0.001
	Food insecure	671 (44.90%)	18.95%–23.07%	37.93 (15.90)	10.50–62.19	
	*Severity of food insecurity*					
	Food secure	824 (55.10%)	52.59%–57.62%	42.49 (15.71)	11.40–63.03	< 0.001
	Mild food insecurity	313 (20.90%)	18.95%–23.07%	39.77 (15.53)	11.49–62.19	
	Moderate food insecurity	239 (16.00%)	14.22%–17.93%	37.77 (15.48)	10.50–60.70	
	Severe food insecurity	119 (8.00%)	6.69%–9.44%	34.92 (16.26)	13.03–59.12	

Abbreviations: CI, confidence interval; IQR, interquartile range; MCS, mental component score; PCS, physical component score; SD, standard deviation.

^a^ Chi‐square test.

Bivariate associations between independent variables and quality of life scores are presented in Table S2.

A backward multiple linear regression was conducted to identify predictors of the physical component score of quality of life (PCS). Male gender, single marital status, nuclear family structure (or having separated parents), higher maternal education, sufficient perceived income, and the absence of chronic disease were all significantly associated with higher PCS scores. Conversely, the presence of food insecurity was independently associated with a significant decrease in physical quality of life scores (*p* < 0.001). These predictors together explain about 10.4% of the variance in physical quality‐of‐life scores. During the elimination process, student's faculty, paternal education, primary source of financial support, current residence, and academic achievement level were sequentially removed (Table 3).

**TABLE 3:** Multiple linear regression analysis predicting SF‐12 physical component score (PCS) of quality of life (*n* = 1.495).

Variables	*B*	SE	*β*	*p*	95% CI
*Gender*					
Female (Ref)	—	—	—	—	—
Male	2.000	0.391	0.128	< 0.001	1.233–2.766
*Marital status*					
Married/Divorced/Other (Ref)	—	—	—	—	—
Single	4.056	1.481	0.073	0.006	1.150–6.961
*Family type*					
Extended (Ref)	—	—	—	—	—
Nuclear	1.694	0.612	0.085	0.006	0.492–2.895
Parents separated	2.087	0.926	0.070	0.024	0.271–3.903
*Perceived income*					
Inadequate (Ref)	—	—	—	—	—
Adequate	2.214	0.454	0.123	< 0.001	1.323–3.104
*Mother's education*					
≤ Middle school (Ref)	—	—	—	—	—
≥ High school	1.507	0.432	0.099	< 0.001	0.660–2.355
*Chronic disease*					
Yes (Ref)	—	—	—	—	—
No	4.077	0.660	0.153	< 0.001	2.782–5.371
*Food insecurity*					
No (Ref)	—	—	—	—	—
Yes	−1.846	0.380	−0.122	< 0.001	(−2.592) to (−1.101)

*Note:* Analysis was conducted using backward linear regression method. Variables included in the initial model: gender, student's faculty, marital status, family type, perceived income status, mother's and father's education level, current living place, chronic disease status, source of financial support, food insecurity status, and academic achievement level. Dependent variable: physical component score (PCS). Model *R* ^2^ = 0.104.

Abbreviations: CI, confidence interval; Ref, reference category; SE, standard error.

The multiple linear regression analysis was conducted to examine factors associated with the mental component score of quality of life (MCS) among university students (*n* = 1.495). In the final step, four variables remained significant. These predictors together explain approximately 5.8% of the variance in mental component scores. During elimination, class year, marital status, paternal education, and current residence were sequentially removed. For mental quality of life, the regression model (Table 4) revealed that sufficient perceived income, non‐smoking status (including former smokers), and good academic achievement were significant positive predictors. Consistent with the physical health findings, food insecurity remained a strong negative predictor, associated with a substantial reduction in MCS scores (*p* < 0.001) (Table 4).

**TABLE 4:** Multiple linear regression analysis predicting SF‐12 mental component score (MCS) of quality of life (*n* = 1.495).

Variables	*B*	SE	*β*	*p*	95% CI
*Perceived income*					
Inadequate (Ref)	—	—	—	—	—
Adequate	3.070	0.638	0.123	< 0.001	1.819–4.320
*Smoking status*					
Current (Ref)	—	—	—	—	—
Former/No	1.689	0.639	0.067	0.008	0.434–2.943
*Academic achievement*					
Low (Ref)	—	—	—	—	—
Good	1.557	0.529	0.074	0.003	0.520–2.595
*Food insecurity*					
No (Ref)	—	—	—	—	—
Yes	−2.736	0.537	−0.131	< 0.001	(−3.790) to (−1.682)

*Note:* Analysis was conducted using backward linear regression method. Variables included in the initial model: gender, year of study, marital status, perceived income status, father's education level, current living arrangement, smoking status, food security status, and academic achievement level. Dependent variable: mental component score (MCS). Model *R* ^2^ = 0.058.

Abbreviations: CI, confidence interval; Ref, reference category; SE, standard error.

## 4 Discussion

The present cross‐sectional study reveals critical insights into the relationship between food insecurity (FI) and quality of life (QoL) among university students. The mean PCS score was 51.44 ± 7.51, and the mean MCS score was 40.07 ± 10.42, indicating a moderate level of perceived physical health but lower perceived mental health among the student population.

Our mean PCS resembles figures reported among Nepali students in South Korea (Bhandari 2012), Chinese undergraduates (Ge et al. 2019), and also in Italian students (Ruotolo et al. 2021), while our MCS is lower than those populations. Mexican students exhibited significantly higher MCS and PCS scores (Núñez‐Rocha et al. 2020). These disparities may stem from cultural, socioeconomic, or methodological differences, such as variations in social support systems or measurement tools. The lower mental health scores in our study underscore the potential need for enhanced mental health support services and interventions within the university environment.

### 4.1 Food Insecurity as a Determinant of QoL

Overall, nearly half of students (44.9%) experienced some level of food insecurity, and these students reported significantly lower health‐related quality of life‐both physically and mentally. Moreover, severity of food insecurity was associated with a clear, stepwise decline in both PCS and MCS scores. The inverse relationship between FI severity and QoL mirrors findings from global studies.

One large multi‐campus study conducted in the USA reported that food‐insecure students experienced more days with poor mental health (Hagedorn et al. 2021). Also, a longitudinal study found that experiencing emergent or persistent food insecurity was associated with significantly higher odds of moderate to severe anxiety and depression (Slotnick et al. 2024). This convergence across different studies suggests a robust relationship between the lack of consistent access to adequate food and poorer mental health among university students. Several potential mechanisms may explain this association. The uncertainty and worry associated with not having enough food can lead to chronic stress and anxiety (Haro‐Contreras et al. 2025). Food‐insecure students often experience psychological distress, including symptoms of depression and feelings of loneliness, which can significantly impact their mental well‐being (Pourmotabbed et al. 2020). Furthermore, feelings of shame and social isolation due to their inability to afford food can also contribute to poorer mental health (Meza et al. 2019).

In addition to its impact on mental well‐being, our study also found a significant decrease in the physical component score (PCS) of quality of life among food‐insecure students. This finding aligns with research by Hagedorn et al. (2021). Several pathways may contribute to this association. Food‐insecure students often have poorer diet quality and experience nutritional deficiencies due to limited resources. The need to prioritize affordability can lead to increased consumption of low‐quality, processed foods that are often nutrient‐poor (Elzein et al. 2017). Reduced energy levels and persistent fatigue resulting from inadequate nutrition can hinder their ability to engage in physical activity, further impacting their physical health (Haro‐Contreras et al. 2025). A notable finding of our study is the clear, stepwise decline in both PCS and MCS scores with increasing severity of food insecurity. This indicates a dose–response relationship, where the negative impact on both physical and mental quality of life intensifies as the lack of access to adequate food becomes more severe. These findings suggest that integrating food security as a priority within university student support frameworks is crucial for enhancing student quality of life.

### 4.2 Gender Differences in Physical Quality of Life

The regression results revealed a significant gender disparity in the physical component score (PCS) of quality of life. Specifically, male students reported higher PCS scores compared to female students. This gender difference aligns with previous research documenting similar patterns. For instance, a study conducted in Sweden found that female university students consistently reported lower health‐related quality of life scores than their male counterparts (Vaez and Laflamme 2003). Similarly, in a comprehensive cross‐national study, Lee et al. (2020) found consistent gender disparities across five diverse countries (China, Ghana, India, Russia, and South Africa) (Lee et al. 2020). Such differences may reflect multifactorial influences, including biological predispositions, sociocultural norms, and health‐related behaviors. Also, sociocultural factors regarding gender norms and health behaviors could influence how students evaluate their physical well‐being. Sociocultural norms emphasizing male stoicism may discourage men from reporting physical limitations, potentially inflating self‐rated PCS scores (Courtenay 2000).

### 4.3 Marital Status and Physical QoL Among University Students

In our study, single university students demonstrated significantly higher physical component scores (PCS) compared to their married, divorced, or widowed peers. Our study specifically focused on university students. This demographic represents a younger population compared to many studies on marital status. Unmarried students may maintain more active social networks, fostering opportunities for physical activity and health‐promoting behaviors. Strong social ties have been linked to better adherence to exercise regimens and healthier dietary patterns (Umberson and Karas Montez 2010). Also, marriage may bring additional responsibilities during university life, reducing the time allocated for physical activity, which may negatively affect physical quality of life. It is important to note that our findings should be interpreted within the university context, where single status represents the demographic norm. The relatively small proportion of married, divorced, or widowed participants in our sample reflects typical university demographics but warrants caution when generalizing results.

### 4.4 Family Structure and Physical Health Outcomes

Regression analysis revealed that family structure is a significant predictor of the physical component score (PCS). Specifically, students residing in nuclear families and those with separated parents exhibit higher self‐perceived physical health. Nuclear families may have more concentrated resources, including financial support, emotional attention, and parental involvement, which can directly contribute to a student's physical well‐being. Furthermore, the structure of nuclear families often centers on the parent–child relationship, allowing parents to directly channel resources, time, and support towards their children's needs (Langton and Berger 2011; Turagabeci et al. 2007). While extended families theoretically could offer a broader network of support, the distribution of these resources might be less direct. While the elevated PCS among students with separated parents introduces a counterintuitive narrative at first glance, it can be explored through several potential lenses. One possible explanation centers on the potential reduction of conflict within the student's immediate environment. For some students, parental separation might lead to a decrease in household conflict, a known significant stressor that can negatively impact physical health (Carrigan‐Smith 2015).

### 4.5 Maternal Education as a Long‐Term Determinant of Physical Well‐Being

Students whose mothers had completed high school education or higher demonstrated significantly better physical component scores (PCS) on quality‐of‐life measures. This positive association between maternal education and physical health outcomes aligns with existing literature (Cárdenas‐Fuentes et al. 2022; von Rueden et al. 2006). The observed association may be mediated by several factors. Higher maternal education is often correlated with greater socioeconomic stability, which facilitates access to nutritious food, safe living environments, and preventive healthcare. Additionally, educated mothers are more likely to model health‐conscious behaviors and advocate for timely medical interventions, thereby fostering long‐term physiological resilience in their children (Prickett and Augustine 2016). Furthermore, mothers with higher education levels have likely navigated the challenges of university life themselves, enabling them to better empathize with and support their children's specific academic and nutritional needs. Our findings extend the significance of early‐life social determinants into the university period.

### 4.6 Chronic Disease and Physical QoL

Our finding that the absence of chronic disease is a strong predictor of higher physical component score (PCS) aligns with extensive evidence demonstrating the detrimental impact of chronic conditions on physical health. A large cross‐sectional study reported lower quality of life scores in university students with chronic diseases, supporting the negative impact of chronic diseases on physical quality of life (Jones et al. 2011). Several mechanisms may explain these observations. Chronic diseases often entail continuous symptoms—such as pain, dyspnea, and fatigue—that directly impair physical function and limit participation in daily activities (Hamilton et al. 2023). Also, in university settings, restricted access to comprehensive healthcare services can exacerbate these effects (Orok et al. 2024).

### 4.7 Income and Quality of Life

Our study underscores the significant association between perceived income sufficiency and both mental (MCS) and physical (PCS) components of quality of life among university students. Participants reporting sufficient income demonstrated notably higher MCS and PCS scores compared to their peers with insufficient income. These results align with a substantial body of literature linking socioeconomic status, including income and financial stability, to various health outcomes (Barakat and Konstantinidis 2023; Nutakor et al. 2023; Seo et al. 2023; Sharkey et al. 2011; Wagner et al. 2025). The observed disparities may be rooted in the psychosocial and material advantages associated with higher income. Financial stability enables access to resources that buffer against stress, such as nutritious food, stable housing, educational opportunities, employment prospects, and healthcare, all of which are foundational to both physical health and psychological resilience (McMaughan et al. 2020; Nutakor et al. 2023).

### 4.8 Tobacco Use and Student Well‐Being

The present study identified a significant association between smoking status and mental health‐related quality of life (MCS) among university students, with current smokers demonstrating markedly lower MCS scores compared to non‐smokers and former smokers. This observation aligns with multiple studies (Milić et al. 2020; Bou‐Hamad et al. 2023; Al‐Kalif et al. 2021; Dağtekin et al. 2020) demonstrating that cigarette smoking detrimentally affects mental health and overall quality of life. Moreover, a review by Goldenberg et al. found a negative association between smoking and quality of life (Goldenberg et al. 2014). University students are in a transitional phase and may be more susceptible to peer influence and experimentation with smoking (Habib et al. 2024). Stress related to academic pressures, social adjustment, and financial concerns can contribute to the perception of smoking as a coping mechanism and to the initiation or continuation of smoking (Sotaquirá et al. 2022). Prioritizing smoking cessation programs—integrated with mental health services—could disrupt the cycle of dependency and psychological morbidity.

### 4.9 The Interplay Between Academic Performance and Quality of Life

Our findings indicate that students reporting higher academic performance demonstrated markedly elevated mental health scores compared to their peers with lower achievement levels. This result is consistent with existing literature that demonstrates a positive relationship between academic factors and student well‐being and quality of life (Li and Zhong 2022; Ramón‐Arbués et al. 2022; Song and Hu 2024). Several potential mechanisms supported by the literature may explain this relationship. One prominent factor is the strengthening of self‐efficacy. Academic success, such as achieving good grades or mastering challenging material, can significantly boost a student's confidence in their academic abilities (Song and Hu 2024). This heightened sense of academic self‐efficacy, the belief in one's capacity to succeed in specific academic tasks, is closely linked to improved mental health. Students with strong academic self‐efficacy tend to experience lower levels of anxiety related to their studies, exhibit greater resilience when facing academic setbacks, and possess a stronger sense of control over their learning experiences (Song and Hu 2024).

### 4.10 Strengths and Limitations of the Study

This study offers several notable strengths that enhance the validity and relevance of its findings. The inclusion of a large sample (*n* = 1495) with proportional allocation across 16 faculties strengthens the reliability of detected associations and mitigates random error. Proportional allocation across 16 faculties further improves generalizability within Pamukkale University's student population, capturing diverse academic and socioeconomic profiles. However, we did not claim statistical representativeness of the entire university population‐ sampling was non‐probabilistic and volunteer‐based, which may introduce selection bias. The observed dose–response relationship between food insecurity severity and declining quality‐of‐life scores lends biological plausibility to the findings, reinforcing their public health significance. However, the findings of this study should be interpreted in light of several methodological limitations. The cross‐sectional design of our study allows us to identify associations between food insecurity and quality of life measures but prevents us from establishing causality or temporal relationships. Food insecurity might impact quality of life, but it is equally plausible that poor quality of life could lead to circumstances contributing to food insecurity. The complex bidirectional nature of this relationship requires longitudinal investigation to fully elucidate the causal pathways. Second methodological limitation of our study stems from the convenience sampling approach which, despite achieving proportional allocation across faculties, introduces potential selection bias that warrants careful consideration. Students who voluntarily participated may differ systematically from those who declined—particularly if individuals experiencing severe food insecurity were less likely to participate due to stigma, time constraints from part‐time employment, or caregiving responsibilities—potentially underrepresenting subgroups at highest risk. The data collection settings in classrooms and social areas (such as cafeterias and libraries) may have further introduced self‐selection bias, as these environments might attract students with specific characteristics, potentially exposing respondents to distractions or peer influence that could increase social desirability bias in their responses. We modified the HFIAS recall window from the original standard (commonly 30 days or 12 months depending on application) to “during your university life” while intended to capture cumulative prevalence which relies on long‐term memory; this change may limit direct comparability with studies using the instrument's original recall period. Also, the SF‐12 utilizes a 4‐week recall period, which differs from the adapted HFIAS recall period. These modifications were designed to enhance relevance for the university student population but may limit direct comparability with other studies using standard protocols. Future research should consider using both standard and modified protocols to enable benchmarking against international findings. Collectively, these sampling limitations affect the generalizability of our findings to the broader university student population, especially to those experiencing the most severe forms of food insecurity, who might be systematically missing from our sample due to the very conditions we aimed to study. Third, all key measures—including sociodemographic characteristics, income status, self‐reported height and weight (for BMI calculation), academic achievement, and chronic disease history—relied on self‐report, which can introduce measurement error and misclassification. Although we collected extensive demographic information, our regression models explained relatively modest portions of the variance in quality‐of‐life scores (10.4% for PCS and 5.8% for MCS). This suggests that other important factors not captured in our study may influence quality of life among university students. Additionally, the exclusion of non‐Turkish‐speaking students limits generalizability to international or linguistically diverse populations. Future research should address these limitations through longitudinal designs, and objective measures (e.g., university records for GPA, clinical BMI assessments). Mixed‐methods approaches could further elucidate how structural factors (e.g., financial aid policies) and individual coping strategies interact to influence student well‐being.

## 5 Conclusion

This study provides compelling evidence of the significant relationship between food insecurity and quality of life among university students at Pamukkale University in Denizli, Türkiye. Nearly half (44.9%) of the students experienced food insecurity, with severity levels ranging from mild to severe. Food‐insecure students exhibited significantly lower physical and mental health scores compared to their food‐secure peers. A dose–response relationship emerged, where escalating food insecurity severity correlated with progressive declines in both physical and mental health scores, underscoring its role as a critical determinant of student well‐being. For physical quality of life, being male, single, living in a nuclear family or with separated parents (compared to an extended family), having a sufficient perceived income, a mother with a high school education or higher, and the absence of a chronic disease were all positively associated with higher scores. For mental quality of life, perceived sufficient income, not being a current smoker or having quit smoking, and good academic achievement were significant positive predictors, alongside food security.

The substantial prevalence of food insecurity and its demonstrable negative impact on both the physical and mental well‐being of university students underscore the urgent need for targeted interventions. Universities should prioritize the establishment or expansion of accessible and destigmatized food support programs, such as food pantries, subsidized meal plans for low‐income students, and partnerships with local food banks. University health and counseling services should incorporate routine screening for food insecurity during student consultations and intake forms. Students screening positive should be provided immediate information on campus resources (food pantry or subsidized meal plans), brief financial counseling, and a referral pathway to campus social services or mental health support. Implementation should be accompanied by staff training to ensure confidentiality and reduce stigma.

## Author Contributions

**Ümmühan İnci Kandemir:** data curation, formal analysis, investigation, methodology, writing – original draft. **Süleyman Utku Uzun:** conceptualization, formal analysis, methodology, supervision, writing – original draft, writing – review and editing.

## Funding

The authors have nothing to report.

## Ethics Statement

The protocol was approved by the Pamukkale University Non‐Interventional Clinical Research Ethics Committee (Approval No. E‐579242; Approval Date: 11 September 2024). All participants provided written informed consent, with assurances of anonymity. Permissions were obtained for all adapted scales.

## Conflicts of Interest

The authors declare no conflicts of interest.

## Supporting information


**Table S1:** Descriptive statistics of SF12 items.

**Table S2:** SF‐12 Physical Component Summary (PCS) and Mental Component Summary (MCS) scores by participants' sociodemographic characteristics—bivariate comparisons (*N* = 1495).

## Data Availability

The data that support the findings of this study are available on request from the corresponding author. The data are not publicly available due to privacy or ethical restrictions.
