# Supplementary material for: The Academic Hunger Gap: From Plates to Well‐Being—How Food Insecurity Undermines Quality of Life in University Students
Source: Food Sci Nutr. 2026 Mar 16;14(3):e71663. doi: 10.1002/fsn3.71663 (PMC13093807; doi:10.1002/fsn3.71663)
Supplement: Supplementary file 1 — Table S1: Descriptive statistics of SF12 items. [file FSN3-14-e71663-s001.docx]

**Supplementary Table S1 - Descriptive Statistics of SF12 Items**

| **SF-12 Domain / Item** | **Response Category** | **n** | **%** |
| --- | --- | --- | --- |
| **1.** **General Health** | Excellent | 97 | 6.5 |
| (In general, would you say your health is:) | Very Good | 438 | 29.3 |
|  | Good | 588 | 39.3 |
|  | Fair | 330 | 22.1 |
|  | Poor | 42 | 2.8 |
| **2. Physical Functioning** | Yes, limited a lot | 38 | 2.5 |
| (Moderate activities, such as moving a table, pushing a vacuum cleaner, bowling, or playing golf) | Yes, limited a little | 295 | 19.7 |
|  | No, not limited at all | 1162 | 77.7 |
| **3.** **Physical Functioning** | Yes, limited a lot | 59 | 3.9 |
| (Climbing several flights of stairs) | Yes, limited a little | 384 | 25.7 |
|  | No, not limited at all | 1052 | 70.4 |
| **4.** **Role-Physical** | Yes | 412 | 27.6 |
| (Accomplished less than you would like as a result of your physical health) | No | 1083 | 72.4 |
| **5.** **Role-Physical** | Yes | 313 | 20.9 |
| (Were limited in the kind of work or other activities as a result of your physical health) | No | 1182 | 79.1 |
| **6.** **Role-Emotional** | Yes | 701 | 46.9 |
| (Accomplished less than you would like as a result of any emotional problems) | No | 794 | 53.1 |
| **7.** **Role-Emotional** | Yes | 714 | 47.8 |
| (Did work or other activities less carefully than usual as a result of any emotional problems) | No | 781 | 52.2 |
| **8.** **Bodily Pain** | Not at all | 735 | 49.2 |
| (How much did pain interfere with your normal work?) | A little bit | 461 | 30.8 |
|  | Moderately | 237 | 15.9 |
|  | Quite a bit | 45 | 3.0 |
|  | Extremely | 17 | 1.1 |
| **9.** **Mental Health** | All of the time | 92 | 6.2 |
| (Have you felt calm and peaceful?) | Most of the time | 384 | 25.7 |
|  | A good bit of the time | 357 | 23.9 |
|  | Some of the time | 504 | 33.7 |
|  | A little of the time | 117 | 7.8 |
|  | None of the time | 41 | 2.7 |
| **10. Vitality** | All of the time | 110 | 7.4 |
| (Did you have a lot of energy?) | Most of the time | 289 | 19.3 |
|  | A good bit of the time | 359 | 24.0 |
|  | Some of the time | 515 | 34.4 |
|  | A little of the time | 190 | 12.7 |
|  | None of the time | 32 | 2.1 |
| **11.** **Mental Health** | All of the time | 111 | 7.4 |
| (Have you felt downhearted and blue?) | Most of the time | 258 | 17.3 |
|  | A good bit of the time | 268 | 17.9 |
|  | Some of the time | 550 | 36.8 |
|  | A little of the time | 266 | 17.8 |
|  | None of the time | 42 | 2.8 |
| **12.** **Social Functioning** | All of the time | 23 | 1.5 |
| (How much of the time has your physical health or emotional problems interfered with your social activities?) | Most of the time | 112 | 7.5 |
|  | Some of the time | 442 | 29.6 |
|  | A little of the time | 497 | 33.2 |
|  | None of the time | 421 | 28.2 |
