# Supplementary material for: The Academic Hunger Gap: From Plates to Well‐Being—How Food Insecurity Undermines Quality of Life in University Students
Source: Food Sci Nutr. 2026 Mar 16;14(3):e71663. doi: 10.1002/fsn3.71663 (PMC13093807; doi:10.1002/fsn3.71663)
Supplement: Supplementary file 2 — Table S2: SF‐12 Physical Component Summary (PCS) and Mental Component Summary (MCS) scores by participants' sociodemographic characteristics—bivariate comparisons (N = 1495). [file FSN3-14-e71663-s002.docx]

| **Supplementary Table S2- SF-12 Physical Component Summary (PCS) and Mental Component Summary (MCS) Scores By Participants’ Sociodemographic Characteristics — Bivariate Comparisons (N = 1,495)** | | | | | |
| --- | --- | --- | --- | --- | --- |
| **Variable** | **Category** | **PCS**  **Mean ± SD** | **p value** | **MCS**  **Mean ± SD** | **p value** |
| **Age** | ≤ 20 years  > 20 years | 51.42 ± 7.45  51.48 ± 7.61 | 0.802 | 39.93 ± 10.35  40.29 ± 10.52 | 0.544 |
| **Sex** | Male  Female | 52.77 ± 7.06  50.71 ± 7.67 | < 0.001 | 41.44 ± 10.78  39.32 ± 10.14 | < 0.001 |
| **Faculty** | STEM / Quantitative Faculties  Humanities / Social Sciences Faculties | 52.12 ± 7.56  51.02 ± 7.47 | 0.002 | 39.67 ± 10.43  40.34 ± 10.42 | 0.253 |
| **Academic Year** | 1st year  Other years | 51.56 ± 7.61  51.40 ± 7.48 | 0.457 | 41.25 ± 10.70  39.59 ± 10.27 | 0.002 |
| **Marital Status** | Single  Married  Divorced / widowed / separated | 51.50 ± 7.49  47.05 ± 8.30  55.47 ± 1.35 | 0.015 | 39.98 ± 10.40  44.76 ± 11.09  44.33 ± 5.41 | 0.046 |
| **Family Type** | Nuclear family  Extended family  Parents separated / other | 51.66 ± 7.51  49.59 ± 7.53  51.67 ± 7.25 | 0.003 | 40.20 ± 10.32  38.88 ± 10.71  40.46 ± 11.18 | 0.223 |
| **Longest-Lived Residence** | City center  District center  Village | 51.44 ± 7.48  51.55 ± 7.60  51.20 ± 7.59 | 0.917 | 40.47 ± 10.62  39.80 ± 9.85  38.59 ± 10.80 | 0.104 |
| **Perceived Household Income Status** | Not enough and have debt  Not enough and no debt  Enough and no savings  Enough and have savings | 48.43 ± 8.87  50.07 ± 7.51  51.49 ± 7.27  52.85 ± 6.96 | < 0.001 | 36.00 ± 10.55  38.23 ± 10.85  39.74 ± 10.24  42.47 ± 9.88 | < 0.001 |
| **Mother's Education** | Middle school or less  High school or higher | 50.59 ± 7.68  52.58 ± 7.15 | < 0.001 | 39.93 ± 10.44  40.27 ± 10.40 | 0.420 |
| **Father's Education** | Middle school or less  High school or higher | 51.08 ± 7.52  51.74 ± 7.51 | 0.042 | 39.39 ± 10.63  40.63 ± 10.23 | 0.025 |
| **Current Living Arrangement** | With family/relatives  Student-shared house  Dormitory | 52.03 ± 6.89  51.84 ± 7.90  50.84 ± 7.68 | 0.028 | 41.47 ± 10.59  40.13 ± 10.51  39.11 ± 10.16 | < 0.001 |
| **Smoking Status** | Current smoker  Non-smoker / former smoker | 50.84 ± 7.77  51.61 ± 7.45 | 0.092 | 38.47 ± 10.83  40.52 ± 10.27 | 0.003 |
| **Chronic Disease** | Yes  No | 47.56 ± 8.91  51.82 ± 7.27 | < 0.001 | 38.51 ± 10.84  40.23 ± 10.37 | 0.102 |
| **Source of Living Expenses** | | | | | |
| **Family Support** | Yes  No | 51.68 ± 7.47  50.45 ± 7.67 | 0.019 | 40.17 ± 10.33  39.69 ± 10.84 | 0.614 |
| **Working While Studying** | Yes  No | 51.05 ± 7.95  51.50 ± 7.46 | 0.690 | 39.39 ± 10.61  40.18 ± 10.40 | 0.374 |
| **Student Loan** | Yes  No | 51.34 ± 7.84  51.46 ± 7.47 | 0.991 | 39.66 ± 11.12  40.14 ± 10.32 | 0.583 |
| **State Scholarship** | Yes  No | 51.35 ± 7.68  51.47 ± 7.49 | 0.973 | 39.05 ± 10.69  40.30 ± 10.35 | 0.080 |
| **Private Scholarship** | Yes  No | 50.31 ± 7.55  51.49 ± 7.52 | 0.222 | 38.54 ± 11.53  40.14 ± 10.37 | 0.336 |
| **Other Scholarship / Funding** | Yes  No | 51.84 ± 7.78  51.44 ± 7.52 | 0.559 | 42.00 ± 9.67  40.04 ± 10.44 | 0.470 |
| **Academic Performance**  **(Self-Reported)** | Good  Low | 51.74 ± 7.32  51.20 ± 7.68 | 0.047 | 41.07 ± 10.25  39.24 ± 10.50 | 0.036 |
| **Physical Activity**  **(IPAQ Categories)** | Inactive  Minimally active  Sufficiently active | 51.12 ± 7.57  51.35 ± 8.29  51.74 ± 7.44 | 0.234 | 39.73 ± 10.09  38.76 ± 10.52  40.44 ± 10.70 | 0.172 |
